# The Shock Index revisited – a fast guide to transfusion requirement? A retrospective analysis on 21,853 patients derived from the TraumaRegister DGU®

**DOI:** 10.1186/cc12851

**Published:** 2013-08-12

**Authors:** Manuel Mutschler, Ulrike Nienaber, Matthias Münzberg, Christoph Wölfl, Herbert Schoechl, Thomas Paffrath, Bertil Bouillon, Marc Maegele

**Affiliations:** 1 Department of Trauma and Orthopedic Surgery, Cologne-Merheim Medical Center, University of Witten/Herdecke, Ostmerheimerstr 200, D-51109 Cologne, Germany; 2 Institute for Research in Operative Medicine, University of Witten/Herdecke, Ostmerheimerstr 200, D-51109 Cologne, Germany; 3 Academy for Trauma Surgery, Luisenstr 58/59, D-10117 Berlin, Germany; 4 Department of Trauma and Orthopedic Surgery, BG Hospital Ludwigshafen, Ludwig-Guttmann-Straße 13, D-67071 Ludwigshafen, Germany; 5 Department of Anaesthesiology and Intensive Care, AUVA Trauma Hospital, Salzburg, Austria; 6 Ludwig Boltzmann Institute for Experimental and Clinical Traumatology, Vienna, Austria; 7 Committee of Emergency Medicine, Intensive Care and Trauma Management of the DGU (Section NIS), Berlin, Germany

**Keywords:** Trauma, Shock, Classification, Vital signs, Shock index, Base deficit, Transfusion

## Abstract

**Introduction:**

Isolated vital signs (for example, heart rate or systolic blood pressure) have been shown unreliable in the assessment of hypovolemic shock. In contrast, the Shock Index (SI), defined by the ratio of heart rate to systolic blood pressure, has been advocated to better risk-stratify patients for increased transfusion requirements and early mortality. Recently, our group has developed a novel and clinical reliable classification of hypovolemic shock based upon four classes of worsening base deficit (BD). The objective of this study was to correlate this classification to corresponding strata of SI for the rapid assessment of trauma patients in the absence of laboratory parameters.

**Methods:**

Between 2002 and 2011, data for 21,853 adult trauma patients were retrieved from the TraumaRegister DGU® database and divided into four strata of worsening SI at emergency department arrival (group I, SI <0.6; group II, SI ≥0.6 to <1.0; group III, SI ≥1.0 to <1.4; and group IV, SI ≥1.4) and were assessed for demographics, injury characteristics, transfusion requirements, fluid resuscitation and outcomes. The four strata of worsening SI were compared with our recently suggested BD-based classification of hypovolemic shock.

**Results:**

Worsening of SI was associated with increasing injury severity scores from 19.3 (± 12) in group I to 37.3 (± 16.8) in group IV, while mortality increased from 10.9% to 39.8%. Increments in SI paralleled increasing fluid resuscitation, vasopressor use and decreasing hemoglobin, platelet counts and Quick’s values. The number of blood units transfused increased from 1.0 (± 4.8) in group I to 21.4 (± 26.2) in group IV patients. Of patients, 31% in group III and 57% in group IV required ≥10 blood units until ICU admission. The four strata of SI discriminated transfusion requirements and massive transfusion rates equally with our recently introduced BD-based classification of hypovolemic shock.

**Conclusion:**

SI upon emergency department arrival may be considered a clinical indicator of hypovolemic shock in respect to transfusion requirements, hemostatic resuscitation and mortality. The four SI groups have been shown to equal our recently suggested BD-based classification. In daily clinical practice, SI may be used to assess the presence of hypovolemic shock if point-of-care testing technology is not available.

## Introduction

Uncontrolled hemorrhage is still one of the leading causes of early death in multiply injured trauma patients [1,2]. The initial assessment and management of hypovolemic shock therefore remains one of the key aspects in trauma resuscitation. For the initial evaluation of circulatory depletion, the American College of Surgeons has defined in its training program Advanced Trauma Life Support (ATLS) four classes of hypovolemic shock. This classification is based upon an estimated percentage blood loss. Furthermore, ATLS suggests corresponding vital signs such as the heart rate, systolic blood pressure and the mental status to allocate each patient to the respective shock class [3]. However, the clinical validity of the ATLS classification of hypovolemic shock has been questioned recently by data from the TARN registry and the TraumaRegister DGU® [4-6]. As an alternative, we have hypothesized that a classification based on the physiological parameter base deficit (BD) would be more appropriate to differentiate the presence and extent of hypovolemic shock in trauma patients. Recently, we introduced and characterized four categories of worsening BD emphasizing that this BD-based classification correlated with higher transfusion requirements, mortality as well as injury severity and discriminated patients at risk for early blood transfusions and death more appropriately than the current ATLS classification of hypovolemic shock [7].

Although point-of-care testing (POCT) can provide BD within minutes, not every emergency department (ED) is equipped with this technology. In the absence of POCT, we alternatively suggest the assessment of the Shock Index (SI). The SI, defined by the ratio of heart rate (HR) to systolic blood pressure (SBP), was first introduced by Allgöwer and Burri in 1967 [8,9]. Although the HR and SBP alone have been shown unreliable in determining the presence of hypovolemic shock [4-6,10-13], their ratio as reflected by the SI has been previously emphasized to serve as a capable measure for hemodynamic instability [14-18] and to risk-stratify patients for transfusion requirements and outcomes [14,15,17]. Subsequently, the SI was suggested as a useful clinical indicator for acute hypovolemia, especially in patients who present with HR and SBP within normal ranges [19,20].

The aims of this study were to characterize four groups of worsening SI based upon a large cohort of multiply injured patients, to report transfusion requirements and outcomes within these four groups, and to compare this SI-based classification in its ability to risk-stratify patients according to their need for early blood product transfusion with our recently introduced BD-based classification of hypovolemic shock.

## Materials and methods

### The TraumaRegister DGU® of the German Trauma Society

The TraumaRegister DGU® (the trauma registry of the German Trauma Society) was initiated in 1993 as an anonymous and standardized documentation of severely injured patients [21]. To date, approximately 600 hospitals are contributing data to this multicenter database. The data collection and documentation are structured and linked to four consecutive time phases: the prehospital phase including initial therapy; the ED and initial treatment until ICU admission; the ICU; and discharge. Detailed information about demographics, mechanism of trauma, vital signs and relevant laboratory findings as well as diagnostic procedures and the therapeutic management are enclosed. Additionally, standardized scoring systems such as the Glasgow Coma Scale [22], the Injury Severity Score (ISS) [23] and the Abbreviated Injury Scale [24] are captured. The TraumaRegister DGU® is approved by the review board of the German Trauma Society and is in compliance with the institutional requirements of its members.

### Inclusion criteria and data analysis

Datasets entered into the TraumaRegister DGU® between 2002 and 2011 were retrieved for analysis. The inclusion criteria for the present study were age ≥16 years, primary admission, and complete datasets for SBP, HR and Glasgow Coma Scale as well as for BD upon ED admission. The SI was calculated for each individual patient by the ratio of HR to SBP.

Based upon previous observations by Zarzaur and colleagues [15], four groups of worsening SI were analyzed. Group I was defined *a priori* by SI <0.6 (no shock), group II by SI ≥0.6 to <1.0 (mild shock), group III by SI ≥1.0 to <1.4 (moderate shock) and group IV by SI ≥1.4 (severe shock). Analyses of vital signs, demographics and injury patterns as well as the therapeutic management such as transfusion rates, administration of fluids and the use of vasopressors were assessed for each SI group. Massive transfusion (MT) was defined by the administration of ≥10 blood products (including packed red blood cells, fresh frozen plasma and thrombocyte concentrates) until ICU admission. Coagulopathy was defined by a Quick’s value ≤70%, which is equivalent to International Normalized Ratio ≥1.3 [25,26]. In accordance with the American College of Chest Physicians/Society of Critical Care Medicine Consensus Conference, sepsis was defined by the presence of a systemic inflammatory response syndrome as a result of a confirmed infectious process [27].

For the comparison of the novel SI-based classification, the four groups of worsening SI were compared with our recently introduced BD-based classification of hypovolemic shock [7]. Patients were therefore classified according to their SI at ED admission and their BD at ED admission. For each classificatory approach, transfusion requirements were compared within the four groups.

### Statistical methods

For continuous variables, data are shown as mean ± standard deviation as well as median and interquartile range. For categorical variables, percentages are presented. Normal distributions were excluded using the Shapiro–Wilk test. As most data were not normally distributed, differences between the four groups of worsening SI were evaluated by Kruskal–Wallis test. Accordingly, categorical variables were analyzed with the chi-square test. For the comparison of SI and BD in the prediction of transfusion requirements, the area under the receiving operating characteristics curve was calculated, with occurrence of transfusion (≥1 blood product) as the state variable. The comparison of two areas under the receiving operating characteristics curve was based upon the 95% confidence interval for each curve. For all statistical analyses, *P* <0.05 was considered statistically significant. All data were analyzed using IBM SPSS (SPSS 19; IBM SPSS, Chicago, IL, USA).

## Results

### Characterization of the four groups of hypovolemic shock based upon the Shock Index at emergency department admission

A total of 21,853 datasets of severely injured patients were identified for further analysis. Worsening of SI category was associated with a higher magnitude of injury, as reflected by corresponding increments in ISS, New Injury Severity Scores and Revised Injury Severity Classification scores (Table 1). The mortality rate increased from 10.9% in group I to 39.8% in group IV. Simultaneously, with worsening of SI category, an increasing incidence of shock-related complications (for example, sepsis and multiple organ failure) was observed. Consequently, overall hospital and ICU length of stays as well as days on a ventilator increased. The analysis of relevant vital signs is depicted in Table 2. Patients with the highest SI (≥1.4) presented with the lowest Glasgow Coma Scale (score of 3 [3]) and had the highest rate of prehospital intubation (80.7%). A higher SI was associated with a significant decline of hemoglobin values and platelet counts. Upon ED arrival, coagulopathy was present in patients with SI ≥1.0 representing groups III and IV (Table 3).

**Table 1:** Patients classified by Shock Index: demographics, injury mechanism and severities as well as outcome parameters

	**Group I (SI <0.6, no shock)**	**Group II (SI ≥0.6 to <1.0, mild shock)**	**Group III (SI ≥1.0 to <1.4, moderate shock)**	**Group IV (SI ≥1.4, severe shock)**
**Demographics**				
*n* (total, %)	6,482 (29.7)	12,097 (55.4)	2,272 (10.4)	1,002 (4.6)
Male	4,858 (74.9)	8,782 (72.6)	1,638 (72.1)	727 (72.6)
Age (years)	50.3 ± 20.4	43.4 ± 19.3	43.2 ± 19.8	44.1 ± 19.2
Blunt trauma	6,069 (96.0)	11,151 (94.5)	2,077 (92.4)	918 (92.4)
**Injury severity**				
NISS (points)				
Mean ± standard deviation	25.1 ± 15.9	26.7 ± 16.0	35.7 ± 17.3	43.2 ± 17.5
Median (IQR)	22 (14 to 34)	24 (17 to 34)	34 (22 to 48)	41 (29 to 57)
ISS (points)				
Mean ± standard deviation	19.3 ± 12.0	21.6 ± 13.3	29.7 ± 15.6	37.3 ± 16.8
Median (IQR)	17 (10 to 25)	20 (12 to 29)	29 (18 to 38)	34 (25 to 48)
RISC (points)	13.6 ± 21.3	12.4 ± 21.5	24.1 ± 29.9	38.8 ± 34.2
AIS head ≥3 points	2,998 (45.9)	4,903 (40.3)	1,106 (48.3)	522 (51.7)
AIS thorax ≥3 points	2,355 (36.1)	5,410 (44.5)	1,343 (58.6)	703 (69.6)
AIS abdomen ≥3 points	495 (7.6)	1,703 (14.0)	621 (27.1)	417 (41.3)
AIS pelvis/extremities ≥3 points	1,399 (21.4)	3,869 (31.8)	1,044 (45.6)	581 (57.5)
**Outcome**				
Mortality	712 (10.9)	1179 (9.7)	525 (22.9)	402 (39.8)
Hospital LOS (days)				
Mean ± standard deviation	17.1 ± 19.6	20.7 ± 22.2	26.1 ± 26.9	25.2 ± 30.4
Median (IQR)	13 (5 to 22)	15 (6 to 27)	20 (6 to 37)	18 (0 to 38)
ICU (days)				
Mean ± standard deviation	7.5 ± 10.6	9.3 ± 12.1	14.0 ± 16.0	15.5 ± 18.9
Median (IQR)	3 (1 to 10)	4 (2 to 13)	9 (3 to 21)	10 (1 to 24)
Ventilator days				
Mean ± standard deviation	4.8 ± 8.9	6.0 ± 10.1	9.8 ± 13.5	11.9 ± 16.1
Median (IQR)	1 (1 to 5)	1 (0 to 8)	4 (1 to 14)	6 (1 to 18)
Multiple organ failure	689 (12.5)	1,567 (14.7)	569 (28.0)	309 (38.2)
Sepsis	353 (6.3)	855 (7.9)	296 (14.3)	178 (21.6)

Data presented as *n* (%), mean ± standard deviation or median (interquartile range (IQR)). *n* = 21,853; *P* <0.001 for all parameters except sex (*P* = 0.003). *AIS* Abbreviated injury scale, *ISS* Injury severity score, *LOS* Length of stay, *NISS* New injury severity score, *RISC* Revised injury severity classification, *SI* Shock index.

**Table 2:** Patients classified by Shock Index: traditional vital signs presented at ED admission and at scene

	**Group I (SI <0.6, no shock)**	**Group II (SI ≥0.6 to <1.0, mild shock)**	**Group III (SI ≥1.0 to <1.4, moderate shock)**	**Group IV (SI ≥1.4, severe shock)**
**Vital signs**				
SBP at scene (mmHg)				
Mean ± standard deviation	136.8 (32.8)	121.9 (29.4)	105.2 (33.1)	92.9 (34.4)
Median (IQR)	138 (120 to 160)	120 (105 to 140)	100 (90 to 120)	90 (70 to 110)
SBP at ED (mmHg)				
Mean ± standard deviation	148.4 (25.6)	124.1 (20.2)	96.9 (16.8)	70.6 (15.7)
Median (IQR)	147 (130 to 160)	120 (110 to 138)	98 (86 to 108)	70 (60 to 80)
HR at scene (beats/minute)				
Mean ± standard deviation	83.0 (19.2)	94.0 (20.6)	103.7 (26.6)	110.5 (31.3)
Median (IQR)	80 (70 to 95)	94 (80 to 105)	105 (90 to 120)	115 (100 to 130)
HR at ED (beats/minute)				
Mean ± standard deviation	73.7 (13.6)	91.3 (15.1)	109.1 (17.9)	122.7 (19.5)
Median (IQR)	74 (65 to 80)	90 (80 to 100)	110 (100 to 120)	120 (110 to 135)
SI at scene (beats/minute)				
Mean ± standard deviation	0.6 (0.2)	0.8 (0.3)	1.1 (0.4)	1.3 (0.5)
Median (IQR)	0.6 (0.5 to 0.7)	0.8 (0.6 to 0.9)	1.0 (1.0 to 1.0)	1.2 (0.9 to 1.6)
GCS at scene (points)	14 (9 to 15)	14 (8 to 15)	11 (4 to 15)	8 (3 to 14)
GCS at ED (points)	13 (3 to 15)	10 (3 to 15)	3 (3 to 12)	3 (3 to 3)
Intubation rate at ED admission	2,515 (39.3)	5,639 (47.4)	1,538 (68.3)	805 (80.7)

Data presented as *n* (%), mean ± standard deviation or median (interquartile range (IQR)). *n* = 21,853; *P* <0.001 for all parameters. *ED* Emergency department, *GCS* Glasgow coma scale, *HR* Heart rate, *SBP* Systolic blood pressure, *SI* Shock index.

**Table 3:** Patients classified by Shock Index: laboratory characteristics

**Laboratory findings**	**Group I (SI <0.6, no shock)**	**Group II (SI ≥0.6 to <1.0, mild shock)**	**Group III (SI ≥1.0 to <1.4, moderate shock)**	**Group IV (SI ≥1.4, severe shock)**
Hemoglobin (g/dl)				
Mean ± standard deviation	12.8 (2.3)	12.3 (2.6)	10.7 (2.9)	9.2 (3.0)
Median (IQR)	13 (12 to 14)	13 (11 to 14)	11 (9 to 13)	9 (7 to 11)
Thrombocytes (tsd/μl)				
Mean ± standard deviation	213 (73)	214 (76)	197 (79)	176 (92)
Median (IQR)	208 (169 to 252)	209 (167 to 256)	194 (143 to 243)	169 (123 to 219)
Quick’s value (%)				
Mean ± standard deviation	86.0 (20.2)	82.4 (21.1)	69.3 (23.7)	57.7 (24.7)
Median (IQR)	90 (77 to 100)	86 (71 to 98)	71 (53 to 88)	56 (40 to 75)
pTT (seconds)				
Mean ± standard deviation	39.8 (10.2)	31.1 (12.9)	38.7 (24.4)	51.4 (34.0)
Median (IQR)	28 (25 to 32)	29 (25 to 33)	32 (27 to 39)	38 (30 to 59)
Lactate (mmol/l)				
Mean ± standard deviation	2.7 (4.7)	3.1 (5.2)	4.6 (7.7)	6.0 (8.4)
Median (IQR)	2 (1 to 3)	2 (1 to 3)	3 (2 to 5)	4 (2 to 7)

*n* = 21,853; *P* <0.001 for all parameters. *IQR* Interquartile range, *pTT* Partial prothromboplastin time, *tsd* Thousand.

### Transfusion requirement within the four groups of worsening Shock Index

**Figure 1:**
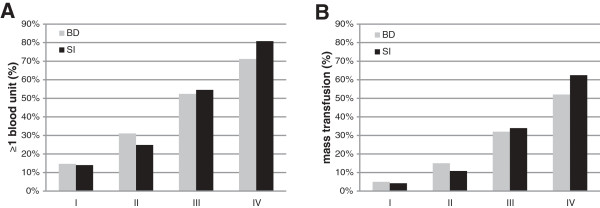
**Transfusion requirements in patients according to classification.** Transfusion requirements in patients classified according to either admission Shock Index (SI; black columns) or according to the base deficit (BD)-based classification of hypovolemic shock (grey columns). **(A)** Percentage of patients with ≥1 blood product. **(B)** Percentage of patients with massive transfusion (≥10 blood units until ICU admission). *n* = 21,853.

An increase in SI category was accompanied by an increasing transfusion requirement (Table 4). On average, the amount of blood units transfused increased from a mean of 1.0 (± 4.8) blood units in group I patients to 21.4 (± 26.2) blood units in group IV patients. As the SI increased above ≥1.0 the percentage of patients who had received ≥1 blood unit between ED arrival and ICU admission increased to 52%, and to 79% in patients with SI ≥1.4 (Figure 1, black columns). Simultaneously, the MT rate was 31% in group III patients and 57% in group IV patients. In contrast, transfusion requirements in groups I and II were significantly lower. The observed transfusion requirement paralleled the predicted transfusion rate as reflected by the Trauma-associated Severe Hemorrhage score from 3.3 (± 3.0) to 15.4 (± 4.9). Furthermore, the amount of fluids administered and the use of vasopressors increased through groups I to IV (Table 4).

**Table 4:** Hemostatic and fluid resuscitation in patients classified by Shock Index

**Transfusion requirements**	**Group I (SI <0.6, no shock)**	**Group II (SI ≥0.6 to <1.0, mild shock)**	**Group III (SI ≥1.0 to <1.4, moderate shock)**	**Group IV (SI ≥1.4, severe shock)**
All blood products/units (*n*)				
Mean ± standard deviation	1.0 (4.8)	2.8 (9.0)	9.9 (17.6)	21.4 (26.2)
Median (IQR)	0 (0 to 0)	0 (0 to 0)	2 (0 to 13)	13 (2 to 31)
pRBC transfusions/units (*n*)				
Mean ± standard deviation	0.8 (2.8)	1.9 (4.9)	5.4 (8.5)	10.7 (12.7)
Median (IQR)	0 (0 to 0)	0 (0 to 0)	2 (0 to 7)	6 (2 to 14)
FFP transfusions/units (*n*)				
Mean ± standard deviation	0.6 (2.4)	1.5 (7.1)	4.4 (8.0)	8.4 (11.1)
Median (IQR)	0 (0 to 0)	0 (0 to 0)	0 (0 to 6)	5 (0 to 12)
TC transfusion/units (*n*)				
Mean ± standard deviation	0.1 (0.5)	0.1 (0.7)	0.6 (2.1)	1.3 (2.5)
Median (IQR)	0 (0 to 0)	0 (0 to 0)	0 (0 to 0)	0 (0 to 2)
TASH score (points)				
Mean ± standard deviation	3.3 (3.0)	5.1 (4.0)	10.3 (4.9)	15.4 (4.9)
Median (IQR)	0 (0 to 0)	4 (2 to 7)	10 (7 to 14)	16 (12 to 19)
Intravascular fluids at scene (ml)				
Mean ± standard deviation	1,092 (745)	1,288 (854)	1,577 (1,126)	1,844 (1,097)
Median (IQR)	1,000 (500 to 1,500)	1,000 (500 to 1,500)	1,500 (1,000 to 2,000)	1,500 (1,000 to 2,500)
Intravascular fluids at ED (ml)				
Mean ± standard deviation	1,716 (1,666)	2,148 (2,490)	3,071 (2,690)	3,955 (3,057)
Median (IQR)	1,000 (500 to 2,000)	1,000 (500 to 2,500)	2,000 (1,000 to 4,000)	3,000 (1,500 to 5,000)
Vasopressors at ED	1,009 (16.5)	2,664 (23.2)	1,064 (48.6)	754 (77.9)

Data presented as *n* (%), mean ± standard deviation or median (interquartile range (IQR)). *n* = 21,853; *P* <0.001 for all parameters. *FFP* Fresh frozen plasma, *pRBC* Packed red blood cells, *TASH* Trauma-associated Severe Hemorrhage, *TC* Thrombocyte concentrates.

### Comparison of the new Shock Index-based classification for hypovolemic shock with our recently suggested base deficit-based classification

The overall accuracy for predicting transfusion requirements was similar for both parameters, as reflected by an area under the receiving operating characteristics curve of 0.711 (0.703 to 0.720) for BD and 0.719 (0.710 to 0.728) for the SI, respectively (*P* = not significant). When the four strata of worsening SI were compared with our recently suggested BD-based classification of hypovolemic shock, the SI discriminated equally the need for early blood product transfusions (Figure 1). Within both approaches, the percentage of patients who received ≥1 blood unit during early ED resuscitation as well as massive transfusion rates increased in a comparable pattern throughout groups I to IV and no clinical relevant differences were observed (Figure 1A,B).

## Discussion

Over the past years, several approaches to assess the extent of hypovolemic shock during early trauma resuscitation have been proposed. Using a combination of vital signs (for example, HR, SBP and mental status) and urinary output, the ATLS classification of hypovolemic shock became one of the most promoted classifications over the last decade. However, the importance and reliability of vital signs to determine the presence and extent of hypovolemic shock have been questioned [4-6,13]. We recently introduced and characterized four classes based on BD representing a modified classification of hypovolemic shock [7]. Although POCT can provide BD within minutes after the patient’s ED admission, not every ED is equipped with this technology. However, one key element of ATLS is its universal and worldwide application, independent of venues and time points of trauma care. In the present analysis, we suggest the SI as an easy accessible and clinical relevant tool to assess the presence of hypovolemic shock in the absence of POCT technology. The four strata of SI discriminated transfusion requirements and MT rates equally with our recently introduced BD-based classification of hypovolemic shock.

There is a growing body of evidence that the ratio of HR to SBP, as reflected by the SI, is the most promising vital sign to detect acute hypovolemia and circulatory failure. A linear relationship between hemorrhage and increasing SI has been described in patients suffering from gastrointestinal bleeding as well as open wounds, and the SI has been shown to correlate best with the quantity of intraperitoneal hemorrhage in ruptured ectopic pregnancies [8,28,29]. Clinically, Rady and coworkers have reported in 275 patients who presented to the ED for urgent medical care that SI >0.9 was associated with the need for immediate treatment and admission of the patient [20]. While HR and SBP still presented within normal limits, these patients displayed reduced central venous oxygen saturation and lactate acidosis, all indicators for the presence of hypovolemic shock [20,30-32]. When healthy blood donors were subjected to a defined blood loss of 450 ml, the SI substantially increased whereas the HR and SBP, taken as separate values, still remained within normal ranges [19]. Due to a negative relationship between SI and left ventricular stroke volume as well as cardiac output, the SI can be used clinically as a fast and noninvasive tool to assess cardiac function during acute hypovolemia [18,31].

In the present study, an increasing SI category reflected injury severity by increasing the ISS as well as higher percentages of thoracic, abdominal and pelvic injuries. Simultaneously, increasing SI paralleled incidences of multiple organ failure and sepsis, all important factors influencing mortality and outcome of trauma patients. In 2,445 trauma patients treated in an urban level I center, SI >0.9 was associated with an increased overall mortality (12.8% vs. 6.1% in patients with SI <0.9). A fivefold increase in mortality was observed in patients with an increase in SI ≥0.3 between the scene of the accident and ED arrival [14]. Zarzaur and colleagues demonstrated that the SI was also a significantly better predictor for 48-hour mortality compared with the SBP and HR. In the overall trauma population as well as in the subgroup of patients aged <55 years, the SI correlated best with transfusion of ≥4 blood units within the first 48 hours after hospital admission [15,16].

The present study emphasizes the role of the SI by demonstrating that the index may discriminate the presence of hypovolemic shock upon ED admission with respect to the need for hemostatic resuscitation and transfusion requirements. To our knowledge, no gold standard has yet been introduced to assess hypovolemic shock including blood loss in trauma, which triggers therapeutic measures and interventions. There is thus currently no option to test our novel approach against a gold standard. The authors have therefore decided to compare this novel approach with our recently introduced and characterized BD-based classification of hypovolemic shock. The assessment of massive transfusion rates and the percentage of patients who received ≥1 blood product, all surrogates for the presence of hypovolemia, clearly demonstrate that the SI can be used equally with our recently suggested BD-based classification of hypovolemic shock in the absence of POCT or laboratory support [7]. Especially, MT rates >30% in group III patients and >50% in group IV patients emphasize this equality. The proposed classification based on the SI may therefore be used as a reasonable clinical alternative if laboratory testing such as POCT is not available.

These results reported here are substantiated by Vandromme and colleagues, who demonstrated that patients with prehospital SI >0.9 have a 1.5-fold increased risk for MT. A further increase of SI >1.3 was even associated with a MT rate of 20% [17]. When the SI at ED admission was assessed, patients with SI between 0.7 and 0.9 had a twofold increased risk for MT and as the SI reached 1.3 or above a 20-fold increase in risk was observed [17].

However, there are some differences between our study and the work by Vandromme and colleagues. When patients were stratified by prehospital SI, a mean ISS of 12.7 for all patients was reported. Even in patients with the highest SI category the mean ISS was 20.3. One should note that in our analysis the mean ISS was already 19.3 in group I patients increasing to 37.3 in group IV patients, and therefore the present study comprises a much more severely injured cohort of patients than all other previous reports. When the frequencies of packed red blood cells as well as massive transfusions were assessed, Vandromme and colleagues had focused on the amounts of blood products administered within the first 24 hours after hospital admission. In contrast, by analyzing the initial phase from ED admission to ICU admission, our study focused primarily on the first hours because this is clinically the most important phase to detect transfusion requirements and to activate blood bank resources and massive transfusion protocols. By inclusion criteria, Vandromme and colleagues had excluded patients with SBP <90 mmHg from their study, which most probably led to elimination of patients with higher transfusion requirements *a priori*. However, the current definition of hypotension as SBP <90 mmHg and a cutoff point for trauma team activation has been questioned. Both in blunt and penetrating trauma and also in the prehospital and in-hospital phase of care, SBP <110 mmHg was already associated with a significant increase in mortality [33-36]. In contrast, there was no restriction on SBP in our study because we intended to propose a classification that can be adopted easily and quickly to all trauma patients upon ED arrival.

In summary, the authors suggest assessing trauma patients in the ED based on the SI if laboratory or POCT devices are not available. Vandromme and colleagues have already proposed that calculation of the SI in the prehospital setting may facilitate the early identification of a relatively high risk for MT and therefore may be incorporated into prehospital triage protocols [17]. Following the ATLS paradigm ‘keep algorithms simple’, the SI may serve as a principle trigger for action in the ED. For group I and group II patients, a careful observation as well as blood typing should be sufficient, unless clinical circumstances dictate otherwise. In group III patients, preparation for transfusion with blood typing and cross-match should be initiated. In group IV patients, where MT rates were >60%, the trauma leader should definitely be prepared for a MT; for example, by activation of a MT protocol and corresponding logistics.

The findings of the present study should be considered in terms of their limitations and strengths. Although, there were only small differences between the vital signs at ED admission and at scene, calculation of the SI at ED admission might be influenced by the prehospital care (for example, the administration of intravenous fluids and/or the use of vasopressors). Furthermore, our trauma registry does not contain information about prior medication such as the use of beta-blockers or antihypertensive agents. Pain and anxiety might also have an influence on SBP and HR and therefore on the SI. Furthermore, the TraumaRegister DGU® comprises, by strict inclusion criteria, only severely injured trauma patients. This may have introduced a selection bias and possibly influenced the accuracy of this classification. Lastly, most patients included in the TraumaRegister DGU® sustained blunt trauma. However, the cardiovascular responses in patients with blunt trauma have been speculated to perhaps differ from those with penetrating injuries. As the percentage of penetrating trauma patients was only marginal in the present analysis, the utility of the four groups of SI was not tested sufficiently in this subgroup. The population of patients seen in this setting is therefore possibly not generalizable. A further validation on penetrating injuries together with a prospective randomized controlled trial to assess the accuracy of our suggested classification is needed.

## Conclusions

The SI upon ED arrival may be considered a clinical indicator of hypovolemic shock with respect to transfusion requirements, hemostatic resuscitation and mortality. The four SI groups have been shown to equal our recently suggested BD-based classification. In daily clinical practice, the SI may be used to assess the presence of hypovolemic shock if laboratory or POCT technology is not available.

## Key messages

• The early recognition and management of hypovolemic shock remains one of the most challenging tasks in the initial assessment of trauma patients.

• Isolated vital signs (for example, SBP and HR) m to have limited reliability in detecting life-threatening hypovolemic shock.

• The SI correlates with the extent of hypovolemia in severely injured patients, as reflected by increased transfusion requirement, higher rates of MT, morbidity and mortality.

• In severely injured patients, the SI-based classification seems to be equivalent to BD with respect to discriminate the need for early blood product transfusion.

• The SI may be considered for early identification of severely injured patients who are at risk for urgent blood transfusion in the absence of laboratory and POCT technology.

## Abbreviations

ATLS: Advanced trauma life support; BD: Base deficit; ED: Emergency department; HR: Heart rate; ISS: Injury severity score; MT: Massive transfusion; POCT: Point-of-care testing; SBP: Systolic blood pressure; SI: Shock index.

## Competing interests

The authors declare that they have no competing interests. This is an unfunded study.

## Authors’ contributions

MMu contributed to the study design, acquisition of data, interpretation and recording of paper. UN and MMü contributed to analysis and interpretation of data and revision of the article. CW, HS, TP and BB contributed to the study design and revision of the article. MMa contributed to the study conception and design, acquisition of data, analysis and interpretation of data, and revision of the article. All authors gave final approval of the current version to be published.
